# Exploring the alteration of gut microbiota and brain function in gender-specific Parkinson’s disease based on metagenomic sequencing

**DOI:** 10.3389/fnagi.2023.1148546

**Published:** 2023-07-12

**Authors:** Minna Zhang, Zhiyuan Zhai, Bo Yang, Le He, Jingyi Wang, Weijie Dai, Liujun Xue, Xiaozhong Yang, Yun Feng, Honggang Wang

**Affiliations:** ^1^Department of Gastroenterology, The Affiliated Huai’an No.1 People’s Hospital of Nanjing Medical University, Huai’an, China; ^2^Department of Neurology, The Affiliated Huai’an No.1 People’s Hospital of Nanjing Medical University, Huai’an, China; ^3^Department of Radiology, The Affiliated Huai’an No.1 People’s Hospital of Nanjing Medical University, Huai’an, Jiangsu Province, China; ^4^Department of Neurology, The Huai’an Clinical College of Xuzhou Medical University, Huai’an, China

**Keywords:** Parkinson’s disease, gut microbiota, fMRI, gender, metagenomic sequencing

## Abstract

**Background:**

The role of the microbiota-gut-brain axis in Parkinson’s disease (PD) has received increasing attention. Although gender differences are known to an essential role in the epidemiology and clinical course of PD, there are no studies on the sex specificity of the microbiota-gut-brain axis in the development and progression of PD.

**Methods:**

Fresh fecal samples from 24 PD patients (13 males, 11 females) were collected for metagenomic sequencing. The composition and function of the gut microbiota were analyzed by resting-state functional magnetic resonance imaging (fMRI). Gender-dependent differences in brain ALFF values and their correlation with microbiota were further analyzed.

**Results:**

The relative abundance of *Propionivibrio*, *Thermosediminibacter*, and *Flavobacteriaceae_noname* was increased in male PD patients. LEfse analysis showed that *Verrucomicrobial*, *Akkermansiaceae*, and *Akkermansia* were dominant in the males. In female patients, the relative abundance of *Propionicicella* was decreased and *Escherichia*, *Escherichia_coli*, and *Lachnospiraceae* were predominant. The expression of the sesquiterpenoid and triterpenoid biosynthesis pathways was increased in male PD patients and was statistically different from females. Compared to the Male PD patients, female patients showed decreased ALFF values in the left inferior parietal regions, and the relative abundance of *Propionivibrio* was positively correlated with the regional ALFF values.

**Conclusion:**

Our study provides novel clinical evidence of the gender-specific relationship between gut microbiota alterations and brain function in PD patients, highlighting the critical role of the microbiota-gut-brain axis in gender differences in PD.

## Graphical Abstract

Figure created with BioRender (https://biorender.com/). 
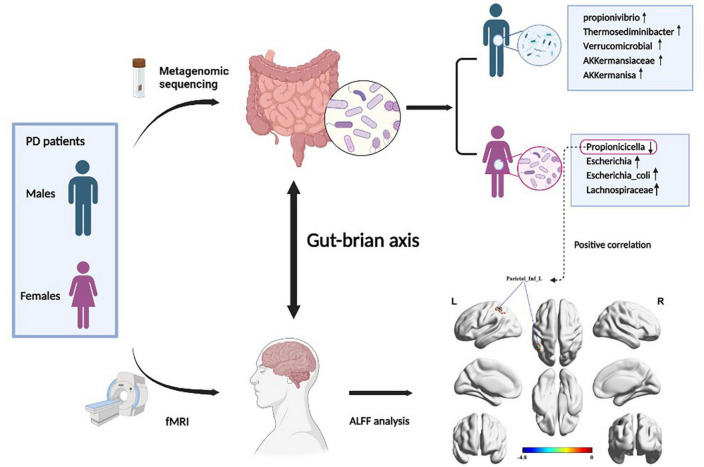

## 1. Introduction

Parkinson’s disease (PD) is the second most common neurodegenerative disease, trailing only Alzheimer’s disease. PD affects middle-aged and elderly individuals, and its key pathological characteristics are the progressive degeneration of nigrostriatal dopaminergic neurons and the formation of Lewy bodies. Epidemiological studies demonstrated that the prevalence of PD in Western countries is as high as 1% over 60, and more than 4% over 80 (Ascherio and Schwarzschild, 2016). The prevalence of PD in China is 1.7% among people over 65 years of age, and it is estimated that the number of Parkinson’s disease patients in China will rise from 1.99 million in 2005 to 5 million by 2030 (Li et al., 2019). The clinical symptoms of PD include mostly progressive movement disorders, including bradykinesia, resting tremor, myotonia, and postural balance disorders. PD is also associated with a variety of non-motor symptoms, such as constipation, sleep disorders, and autonomic dysfunction (Ascherio and Schwarzschild, 2016). As the disease progresses, not only the quality of the patient’s life deteriorates, but also a huge burden on society and families is created.

Increasing evidence points to significant gender-related differences in the epidemiology and clinical features of PD. Although the incidence of PD is twofold higher in males than in females, the female sex is associated with higher mortality and more rapid disease progression (Baldereschi et al., 2000; Dahodwala et al., 2018). Additionally, female patients differ from males in their response to treatment and self-assessment of the quality of life (Cerri et al., 2019). Therefore, biological sex differences have been attributed as one of the important factors influencing the development of PD disease. In addition to the effects of physiological sex, patients with PD exhibited unique changes in gut microbiota, and alterations in gut microbiota composition might be involved in the pathogenesis of PD. Gut microbiota was thought to be a key mediator of bidirectional communication between the gut and the brain along the gut-brain axis (Quigley, 2017). In a study, *Prevotellaceae*, *Lachnospiraceae*, and *Faecalibacterium* were found to be significantly decreased in PD patients compared to healthy controls, and the investigators speculated that these generals may exert anti-PD effects. In addition, the abundance of *Verrucomicrobiaceae*, *Bifidobacteriaceae*, *Christensenellaceae*, and *Ruminococcaceae* was observed to decrease in PD patients (Wallen et al., 2020). Notably, Gender has been shown to influence the complexity and diversity of the gut microbiota, and the gut microbiota was gender-specific in the neurobiology of mental disorders (Clarke et al., 2013; Vemuri et al., 2019). However, no studies have yet elucidated whether there are gender differences in gut microbiota in female and male PD patients.

Pathological studies have shown that PD lesions are not limited to the substantia nigra, but gradually extend to multiple brain regions including the limbic system and extensive neocortex. The progressive development of lesions results in the formation and accumulation of Lewy vesicles in local neurons, neuronal necrosis and loss, and subsequent functional abnormalities in the corresponding brain regions (Lees, 2012). Functional changes in the brain of PD patients can be studied by functional magnetic resonance imaging (fMRI). fMRI provides insights into the mechanisms of damage associated with many clinical symptoms of PD, and helps to understand the mechanisms of neuroplasticity involved in the effectiveness of pharmacological and neurorehabilitation treatments. The Amplitude of Low-Frequency Fluctuations (ALFF) measures blood oxygen level-dependent (BOLD) signals in the low-frequency range that can be used to evaluate the regional neural activity and directly observe changes in brain activity. In PD patients, ALFF can be used to assess neuronal activity in several brain regions, including frontal, parietal, temporal, dorsal thalamus, caudate nucleus, and other regions. In addition, ALFF can be used to determine the severity of motor and non-motor symptoms in patients.

Based on the concept of the gut-brain axis, the present study combines the metagenomic analysis of gut microbiota with fMRI results to investigate whether gender differences in gut microbiota are present in PD patients and whether they correlate with brain activity. Taken together, this study will help to better investigate the role of gut microbiota in the pathogenesis and pathological changes of PD disease.

## 2. Materials and methods

### 2.1. Participants

We recruited 24 age-, weight-, and Hoehn-Yahr (HY) score-matched patients (including 13 males and 11 females) with PD from the Department of Neurology of the First People’s Hospital of Huai’an City. PD was diagnosed according to the primary Parkinson’s disease diagnostic criteria [MDS 2015 criteria (Postuma et al., 2015)], and patients continued stable-dose PD treatment for the duration of the study. The age range of the PD patients was 52–78 years old. Participant exclusion criteria included: Parkinson’s syndrome; chronic gastrointestinal disease; autoimmune disease with gastrointestinal involvement; malignancy; and a history of antibiotic use in the last month. We obtained written informed consent from each patient before sample collection. All patients were assessed on the Body mass index (BMI), UPDRS III (Asakawa et al., 2016), HAMA (Hamilton, 1959), and HAMD (Hamilton, 1967) scales before sample collection. At the same time, we also calculated and counted the L-dopa equivalent dose of each PD patient. The study protocol was approved by the ethics committee of the Affiliated Huai’an No.1 People’s Hospital of Nanjing Medical University.

### 2.2. Fecal sample collection

Fresh stool samples were collected from all PD patients early in the morning before medication was administered. One clinical fecal sample per patient was collected. All samples were stored at −80°C until DNA extraction.

### 2.3. Metagenomic sequencing of the gut microbiota

DNA from 24 fecal samples was extracted using The E.Z.N.A.^®^ Stool DNA Kit (D4015-02, Omega, Inc., USA) according to the manufacturer’s instructions. The total DNA was eluted in 50 μl of Elution buffer by a modification of the procedure described by the manufacturer (QIAGEN) and stored at −80°C until measurement in the PCR by LC-BIO TECHNOLOGIES (HANGZHOU) CO., LTD., Hang Zhou, Zhejiang Province, China. The quality of DNA extraction was determined by agarose gel electrophoresis, while DNA was quantified by UV spectrophotometer. DNA library was constructed by TruSeq Nano DNA LT Library Preparation Kit (FC-121-4001). DNA was fragmented by dsDNA Fragmentase (NEB, M0348S) by incubating at 37°C for 30 min. After the libraries passed quality control, high-throughput sequencing was performed with NovaSeq6000, and the sequencing mode was PE150. The raw data obtained from sequencing were subjected to further analysis. First, sequencing adapters were removed from sequencing reads using cutadapt v1.9. Secondly, low-quality reads were trimmed by fqtrim v0.94 using a sliding-window algorithm. Thirdly, reads were aligned to the host genome using bowtie2 v2.2.0 to remove host contamination. Once quality-filtered reads were obtained, they were *de novo* assembled to construct the metagenome for each sample by IDBA-UD v1.1.1. All coding regions (CDS) of metagenomic contigs were predicted by MetaGeneMark v3.26. CDS sequences of all samples were clustered by CD-HIT v4.6.1 to obtain unigenes. Unigene abundance for a certain sample was estimated by TPM based on the number of aligned reads bybowtie2 v2.2.0. Unigenes were obtained after filtering low abundance expression; Unigenes were compared with NR_mate library to obtain species annotation information using DIAMOND software; Unigenes were compared with GO and KEGG databases for functional annotation. Alpha diversity and beta diversity were determined by the QIIME2, and the pictures were drawn by R (v3.5.2). Liner discriminant analysis (LDA) effect size (LEfse) analyses were performed with the LEfse tool.^1^

### 2.4. MRI data acquisition

Magnetic resonance images were acquired by a 32-channel 3.0-T MRI scanner (Philips, ingenia 3.0 CX), The T1-weighted three-dimensional images were obtained with the following parameter settings: repetition time (TR) = 6.6 ms, echo time (TE) = 3.0 ms, thickness = 1.0 mm, flip angle = 8°, the field of view (FOV) = 240 mm × 240 mm, matrix = 240 × 240; Functional MR images were acquired across 250 scans with a gradient echo EPI sequence: TR = 2,000 ms, TE = 30 ms, and flip angle = 90°. A total of 33 slices (FOV = 230 mm × 230 mm, matrix = 96 × 94, slice thickness = 3.6 mm, and 250 volumes) aligned along the anterior cingulate and posterior cingulate cortex line were acquired. During the rs-fMRI scans, all participants kept their eyes closed, relaxed, motionless, awake, and of nothing. Routine brain fluid-attenuated inverse recovery (FLAIR) sequence scanning was acquired to exclude other cerebral abnormalities.

### 2.5. Image pre-processing

The functional Resting-State fMRI image preprocessing was performed with the software DPASF.^2^ The initial 10-time points were removed to eliminate interference from early detection, Remaining 240-time points were analyzed as described below: slice-timing, realigning for checking head motion (head motion >1.5 mm translation or rotation >1.5° would be excluded), and co-registered the functional MRI images to participants’ 3D-T1 images, then the T1 structural images were segmented into gray matter, white matter and cerebrospinal fluid and then spatially normalized to the Montreal Neurological Institute (MNI) space, the final functional images of normalization matrix were smoothed with a 6 mm (FWHM) Gaussian kernel. Following the filtering of imaging data, linear regression was used to remove spurious variance in head motion, CSF signal, and white matter signal.

### 2.6. The amplitude of low-frequency fluctuations (ALFF) analysis

Firstly, the acquired images were linearly de-trended. After filtering at 0.01–0.08 Hz, the data will be converted into a power spectrum by fast Fourier transform, then calculating the square root of the power spectrum and extracting the ALFF value. The ALFF value of each voxel was divided by the global mean ALFF (mALFF) value to obtain a standardized ALFF value. An independent two-sample *t*-test was used to calculate the ALFF differences between the two groups with the covariate of the head motion parameter. The results were corrected by the Gaussian Random Field theory correction (GRF) (voxel *P*-value < 0.001, cluster *p*-value < 0.05). The peak voxel MNI coordinate of the significant voxel was picked as ROI. The software tool–Rest^3^ was used to extract the ALFF value of the peak voxel for subsequent statistical analysis.

### 2.7. Statistical analysis

The clinical characteristics of the participants were analyzed using the SPSS 26.0 statistical package (IBM SPSS Statistics). Categorical data were calculated using the chi-square test and quantitative data were calculated using independent samples *t*-test (two-tailed). *p* < 0.05 was considered statistically different. Results are shown as mean ± standard error. Correlation analysis was performed using Spearman correlation analysis in SPSS.

## 3. Results

### 3.1. Clinical characteristics of PD patients

A total of 24 patients with PD were enrolled in this study and divided into two groups, including 13 males (PD_M) and 11 females (PD_F). There were no statistically significant differences in age, disease duration, weight, Hoehn-Yahr stage, HAMA, HAMD, and Levodopa equivalent dose between the two groups (*p* > 0.05). However, female patients had higher BMI than males (*p* = 0.001) (Table 1).

**TABLE 1:** Clinical characteristics of PD patients of different genders (mean ± SD).

	M-PD (*n* = 13)	F-PD (*n* = 11)	*P*-value	*t*
Ages, year-old	64.85 ± 7.41	67.09 ± 6.76	0.450	−0.769
Disease duration, year	5.23 ± 4.92	4.00 ± 2.97	0.476	0.724
Weight, kg	62.62 ± 8.92	66.91 ± 8.24	0.237	−1.216
BMI, kg/m^2^	21.57 ± 2.80	26.10 ± 2.97	0.001	−3.843
Hoehn-Yahr	1.92 ± 0.86	2.04 + 0.98	0.342	−0.971
UPDRS III	30.62 ± 17.06	36.73 ± 13.02	0.749	−3.240
HAMA	10.92 ± 7.71	11.73 ± 7.29	0.797	−0.261
HAMD	13.92 ± 9.73	13.82 ± 6.92	0.976	0.030
L-dopa equivalent dose, mg	619.69 ± 228.76	615.05 ± 209.48	0.959	0.052

### 3.2. Sequencing data and gut microbiota diversity

**FIGURE 1:**
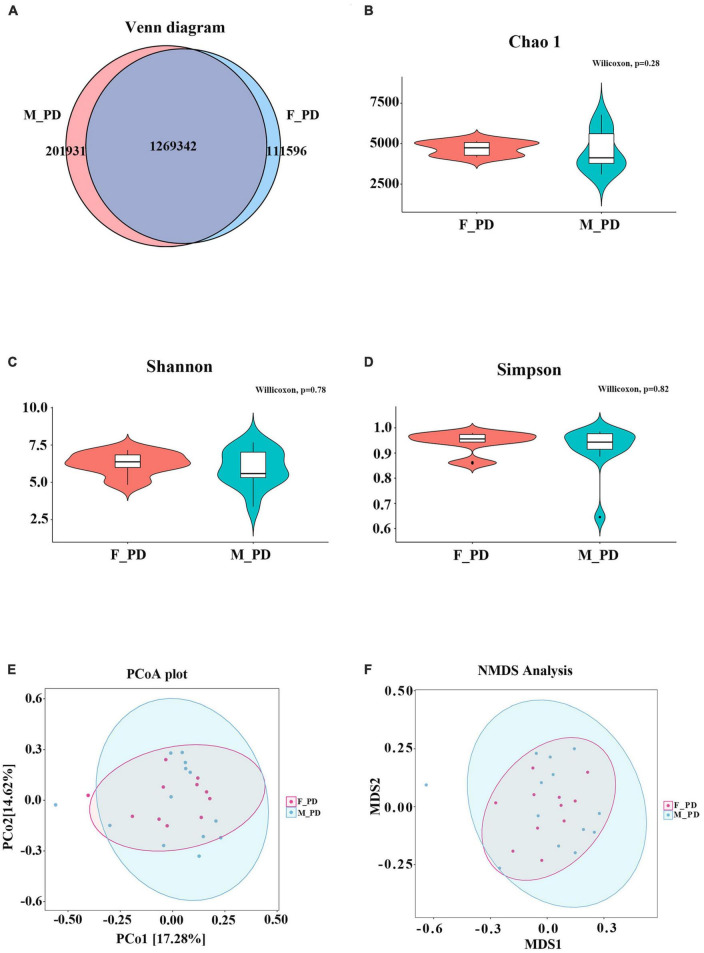
Sequencing data and gut microbiota diversity. **(A)** Venn diagram of Unigenes in different groups. **(B)** Chao index **(C)** Shannon index **(D)** Simpson index **(E)** PCoA score plot **(F)** NMDS analysis.

Metagenomic sequencing was performed on 24 fecal samples collected from PD patients. The Venn diagram illustrates the number of unigenes shared between males and females or unique to one gender (Figure 1A). Specifically, 201,931 unigenes were unique to the PD_M group, and 111596 unigenes were unique to the PD_F group. The total number of unigenes in both groups was 126934. Subsequently, the distribution and composition of the microbial communities in the samples were obtained by species annotation based on sequence information from UniGene. To assess differences in microbiota composition between the two groups, Chao1 (*p* = 0.28), Shannon (*p* = 0.78), and Simpson (*p* = 0.82) indices were used to evaluate the α diversity of gut microbiota (Figures 1B–D), and PCoA and NMDS were used to evaluate the β diversity (Figures 1E, F). However, neither they α nor the β diversity differed significantly between the two groups (*p* > 0.05).

### 3.3. Alterations of gut microbiota composition

**FIGURE 2:**
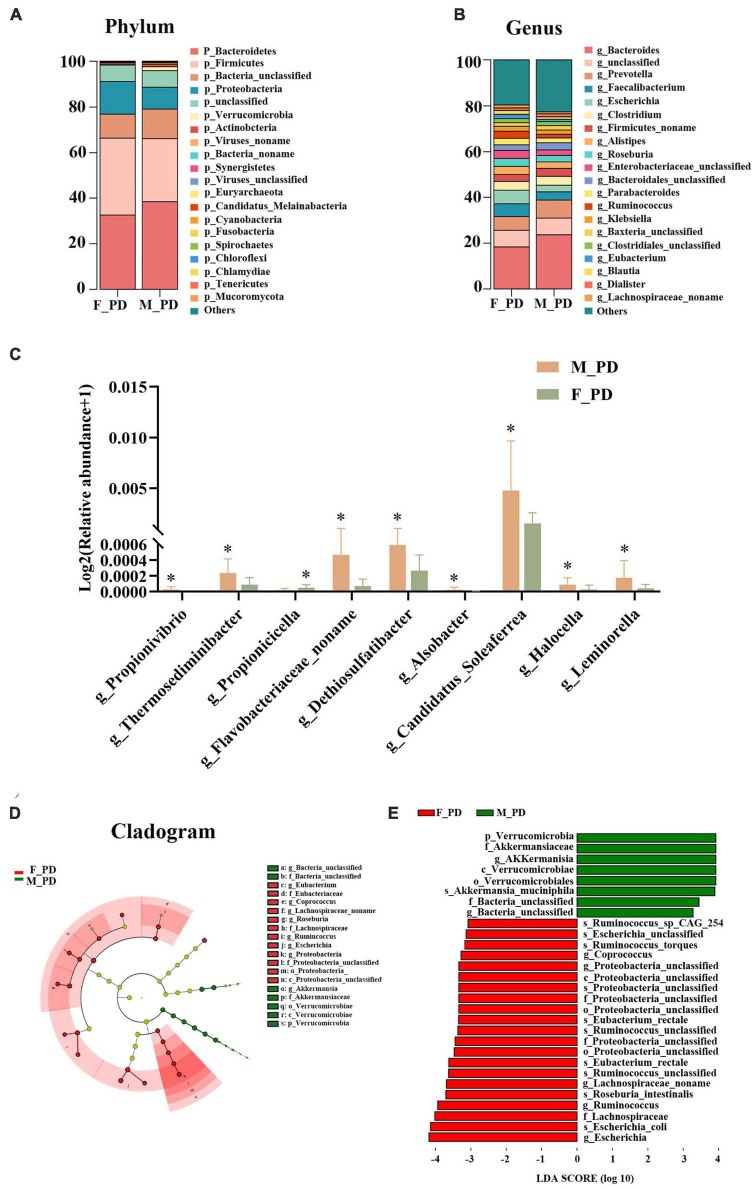
Alterations of gut microbiota composition. **(A)** Bar chart of gut microbiota at the phylum level. **(B)** Bar chart of gut microbiota at the genus level. **(C)** Relative abundance of discriminative gut microbiota at the genus level; *P < 0.05. **(D)** Cladogram generated by LEfSe analysis. **(E)** Liner discriminant analysis (LDA) effect size (LEfSe) analysis.

At the phylum level, gut microbiota in both male and female PD patients were dominated by Bacteroidetes, Firmicutes, and Proteobacteria (Figure 2A). However, the abundance of all phyla did not differ significantly between the two groups. The top three dominant bacteria at the genus level were *Bacteroides*, *Prevotella*, and *Faecalibacterium* (Figure 2B). Next, nine significantly different genera in the two groups were selected by abundance variation analysis (*p* < 0.05 and | log2(fold_change)| > 1). This analysis demonstrated that the relative abundance of *Propionivibrio* (*p* = 0.016), *Thermosediminibacter* (*p* = 0.018), *Flavobacteriaceae_noname* (*p* = 0.033), *Dethiosulfatibacter* (*p* = 0.036), *Alsobacter* (*p* = 0.037), *Candidatus_soleaferrea* (*p* = 0.038), *Halocella* (*p* = 0.045), and *Leminorella* (*p* = 0.049) was increased in the male and decreased in the female PD patients. Conversely, the relative abundance of *Propionicicella* (*p* = 0.031) was decreased in males and increased in females (Figure 2C). To further identify the significant differences between the two groups, the LEfSe analysis was performed for the seven taxonomic strata according to different comparison groups (LDA > 3.0 and *p* < 0.05), and species with significant differences were presented by evolutionary branching plots (Figure 2D) and distribution histograms (Figure 2E). The results demonstrated that the relative abundance of *Verrucomicrobial*, *AKKermansiaceae*, and *AKKermanisa* was increased in the male PD patients. Conversely, the relative abundance of *Escherichia*, *Escherichia_coli*, and *Lachnospiraceae* was significantly higher in the female PD patients.

### 3.4. Functional characterization of differentially expressed genes (DEGs)

**FIGURE 3:**
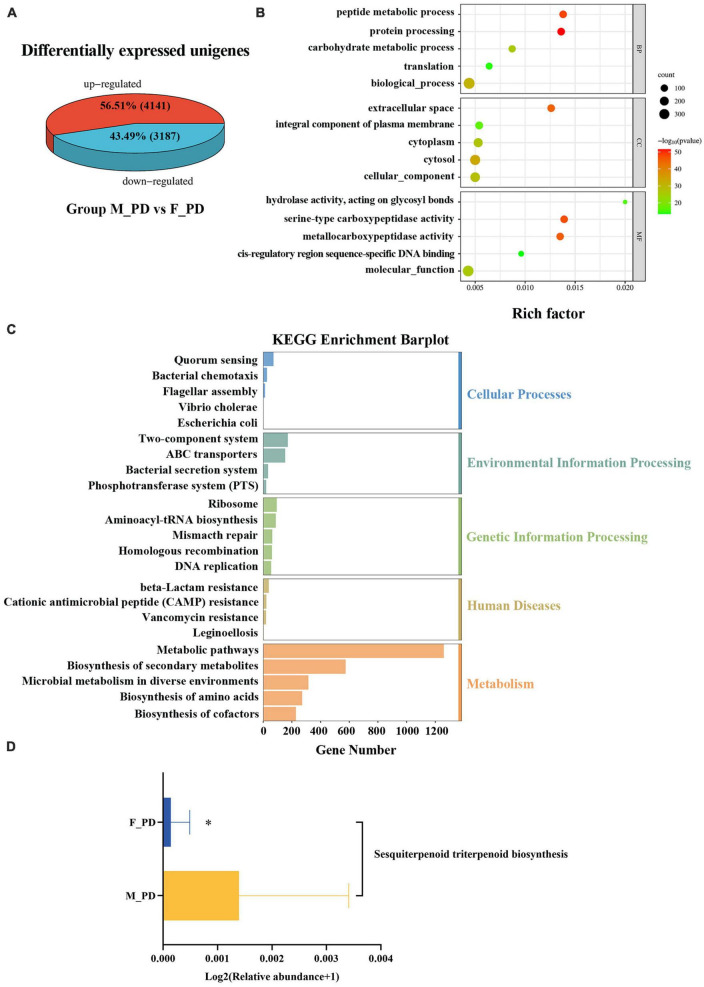
Functional characterization of differentially expressed genes (DEGs). **(A)** Differentially expressed unigenes. **(B)** Bubble plots of GO enrichment analysis. **(C)** Bar plot of KEGG pathway enrichment. **(D)** Expression KEGG pathways with differences in the two groups. *P < 0.05.

The UniGene differential expression analysis showed that the expression of 4141 unigenes were upregulated and the expression of 3187 unigenes expressions were downregulated in both groups (Figure 3A). Next, GO enrichment analysis of DEGs was performed to performed (Figure 3B), and significant DEGs were classified into three main categories: biological process (BP), cellular component (CC), and molecular function (MF). The top three significantly upregulated DEGs in the BP category were related to the peptide metabolic process, protein processing, and carbohydrate metabolic process. In the CC category, the upregulated genes were significantly involved in extracellular space, and an integral component of the plasma membrane. For the category of MF, the upregulated DEGs were correlated with the hydrolase, acting on glycosyl bonds, serine-type carboxypeptidase activity, and metallocarboxypeptidase activity. KEGG pathway enrichment analysis showed significant enrichment in the categories of Cellular Process, Environmental Information Processing, Genetic Information Processing, Human Disease, and Metabolism (Figure 3C). The most significantly enriched pathway was the Metabolic pathways in the Metabolism category. In addition, the upregulation of Sesquiterpenoid and Triterpenoid Biosynthesis in male PD patients was statistically significant (*p* < 0.05) (Figure 3D).

### 3.5. Relationship between the ALFF values of peak brain region and microbial abundance

**FIGURE 4:**
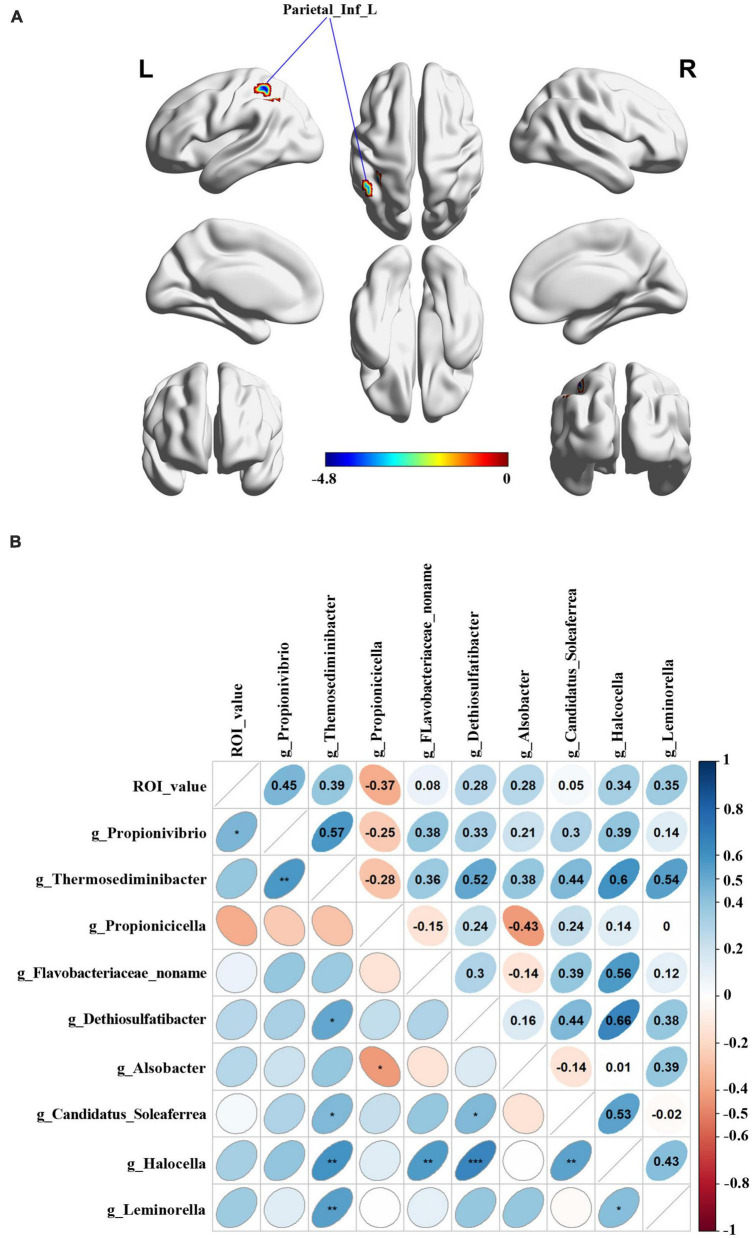
Relationship between the Peak value of ROI analysis and microbial abundance. **(A)** Differential brain functional areas in PD patients by gender. **(B)** Spearman analysis was performed for correlation analysis.

The brain ALFF analysis was performed using the fMRI data obtained in 13 male and 11 female PD patients. Compared to the male group, females exhibited decreased ALFF signals in the left angular gyrus of the parietal inferior margin (Parietal_Inf_L), the left superior parietal gyrus and the left post-central gyrus (Figure 4A). Parietal_Inf_L was selected as the peak brain region and regional ALFF values were extracted for each subject. Spearman correlation analysis between the extracted ALFF values and nine significantly differential gut microbiota (Figure 4B) demonstrated that the abundance of *Propionivibrio* was positively correlated with the ALFF values (*r* = 0.45, *p* = 0.027).

## 4. Discussion

The microbiota-gut-brain axis is considered a complex bidirectional signaling pathway between the gut and the central nervous system, including the autonomic nervous system, immune system, chemotransmitters, and endocrine hormones (Margolis et al., 2021). Recent evidence suggested that the interaction between sex specificity and gut microbiota may play a role in the development of neurodegenerative diseases by influencing the gut-brain axis (Rizzetto et al., 2018; Jaggar et al., 2020). The pathology of intestinal origin of PD was first proposed by Del Tredici and Braak (2008) and was subsequently confirmed by a growing number of human and experimental studies (Lionnet et al., 2018). Gut microbiota can affect the onset and development of PD through multiple mechanisms such as neural pathways, altering host metabolism, modulating peripheral immunity, and influencing drug efficacy (Zhu et al., 2022). While sex hormones, mainly estrogen, were thought to support neuronal antioxidant maintenance of the dopaminergic nervous system (Zarate et al., 2017), thus far, no studies explored sex-specific effects on the onset and progression of PD via the microbiota-gut-brain axis. Based on these premises, the current study further investigated the association between the sex specificity of gut microbiota and correlation with brain function in PD patients.

The results of our study showed no significant difference in gut microbiota diversity between male and female PD patients. Previously, a European study reported a higher α-diversity of gut microbiota in females than in males (Mueller et al., 2006). However, the findings in Asia differ from those in the West, with no significant differences in α-diversity between males and females documented in a Japanese study (Takagi et al., 2019). Additionally, it has been suggested that the association between sex and α-diversity is stronger in young adults than in middle-aged adults, and the most significant changes in gut microbiota diversity occur in early childhood (Yatsunenko et al., 2012). Given that no differences in α-diversity were found between middle-aged people and those in their 70 s (Biagi et al., 2010), we hypothesized that the comparable gut microbiota diversity between male and female PD patients might be related to the average age of the enrolled patients, around 60–70 years. Importantly, our study identified significant differences in the relative abundance of gut microbiota at the genus level between male and female PD patients. We determined that the relative abundance of *Propionivibrio*, *Thermosediminibacter*, *Flavobacteriaceae_noname*, *Dethiosulfatibacter*, *Alsobacter*, *Candidatus_Soleaferrea*, *Halocella*, and *Leminorella* was higher in the male than in the female PD patients. Moreover, the LEfse analysis showed that *Verrucomicrobial*, *Akkermansiaceae*, and *Akkermansia* were dominant in the males. Several studies have shown a significant increase in the levels of *Verrucomicrobial*, *Akkermansiaceae*, and *Akkermansia* in PD patients compared to healthy individuals (Bullich et al., 2019; Shen et al., 2021). A study by Lin and coworkers documented an increased abundance of *Verrucomicrobia* in PD patients that correlated with disease severity, and increased plasma IFN-γ concentrations (Lin et al., 2019). *Akkermansiaceae* is the second family of warty microflora, and *Akkermansia* is the only well-known genus within the family. *Akkermansia* was isolated from the outer mucus layer attached to intestinal epithelial cells and can be involved in the degradation of mucin (Geerlings et al., 2018). Increased abundance of *Akkermansia* in the stool of PD patients has also been detected in many studies (Keshavarzian et al., 2015; Heintz-Buschart et al., 2018; Lin et al., 2019). It has been suggested that *Akkermansia* may be involved in the pro-inflammatory process of PD, causing intestinal barrier disruption and abnormal aggregation of α-synuclein in the enteric nervous system (ENS), accelerating the progression of PD (Fujio-Vejar et al., 2017). The present investigation demonstrated that the relative abundance of *Propionicicella* was decreased in female patients, while *Escherichia*, *Escherichia_coli*, and *Lachnospiraceae* were predominant. The increase in the abundance of *Enterobacteriaceae* was considered essential in intestinal flora disorders associated with PD (Li et al., 2017). The Finnish team Scheperjans et al. first suggested in 2015 that an increased abundance of *Enterobacteria* in PD patients was associated with postural impairment and gait instability (Scheperjans et al., 2015). Conversely, *Lachnospiraceae* is a potentially beneficial family of gut microbiota found in most healthy people (Sorbara et al., 2020). Members of *Lachnospiraceae* produce short-chain fatty acids that play a role in anti-inflammatory reactions and coordinate gastrointestinal nervous system function (Lin et al., 2018; Oliphant et al., 2021). Keshavarzian et al. (2015) showed that the abundance of *Lachnospiraceae* decreased with the duration of PD.

Interestingly, estrogen has recently been reported to affect the gut microbiota. In a cross-sectional study, urinary estrogen levels correlated with fecal microbiota abundance and α-diversity in men and postmenopausal women, and β-glucuronidase production by certain fecal flora was negatively associated with the level of fecal estrogen (Flores et al., 2012). In our study, baseline clinical characteristics indicated that female patients had a higher BMI than male patients. A Chinese study of subjects with different BMI values showed that higher α-diversity was present underweight patients, but no significant diversity differences were observed among obese, normal weight, and overweight patients (Gao et al., 2018). Obese Chinese individuals was characterized by increased *Fusobacterium* in men, whereas women exhibited an increased abundance of *Bifidobacterium*, *Coprococcus*, and *Dialister genera*. Although some of the female patients in our study were overweight, the collected data do not prove the notion that changes in gut microbiota in PD patients of different genders are affected by BMI values; a larger sample size is needed to explore the relationship between BMI and intestinal flora female PD patients.

The metagenomic sequencing results also revealed that differentially expressed unigenes were significantly enriched in protein process, serine-type carboxypeptidase activity, and extracellular space. This information provides direction for further mechanistic investigations. PD is considered a classical “protein disease” in which proteins misfold to form fibrillar aggregates rich in β-fold. Additionally, the results of KEGG enrichment analysis suggested that metabolic pathways may play an important mechanistic role in gender-specific differences in the gut microbiota of PD patients, but metabolomics-related studies were not performed here. These studies, utilizing larger sample sizes, are needed to elucidate the mechanisms underlying gender differences in the gut microbiota of PD patients. In addition, we found that the expression of sesquiterpenoid and triterpenoid biosynthesis pathways was increased in male PD patients and was statistically different from female patients. In this regard, the triterpenoids found in Centella asiatica have been shown to have a neuroprotective function (Gray et al., 2018). For example, Centella asiatica and Centella asiatica glycosides found in Centella asiatica have been shown to have neuroprotective effects in stroke models, reducing cytotoxic damage and microglia activation (Chen et al., 2014). Since it is well known that estrogen plays a protective role in female patients with PD, the possibility that gender-specific gut microbiota plays a protective role in the development of PD warrants additional mechanistic studies.

Gender differences are also present in the human brain. Males have larger total brain volume, cortical surface area and sulcal gyrus, a greater white-to-gray matter ratio, and less dense gray matter than females; these differences may be genetically determined. It has been found that hippocampal plasticity receives sex-related alterations in the microbiome (Darch et al., 2021). Experiments in germ-free mice revealed that alterations in microbiota might indeed lead to changes in dendritic signaling integration in hippocampal circuit regions (Luczynski et al., 2016). All these studies imply that sex differences in the gut-brain axis may be modified by the microbiome. The work by Siani et al. (2017) demonstrated that ovariectomy increases dopaminergic loss in female mice, while supplementation with estrogen prevents dopaminergic loss. This result suggested that sex hormone therapy may benefit PD patients through the gut-brain axis. Sex hormone therapy may bring potential efficacy to PD patients through the gut-brain axis. By ALFF analysis, we identified Parietal_Inf_L, a region of difference in brain function between male PD patients and female PD patients. As previously shown, this brain region is associated with AD-related Aβ alterations and is thought to be one of the potentially A toxic oligomer seeds in the brain (Jiang et al., 2015). In addition, we found that the relative abundance of *Propionivibrio* was positively correlated with the peak value in Parietal_Inf_L. Therefore, we hypothesized that sex-specific changes in intestinal flora abundance might affect brain function in PD patients through the action of the gut-brain axis. Although we have revealed the effects of sex differences on brain and gut microbiota in PD patients, the mechanisms of these interactions and their mutual relationship remain unclear. More experiments are needed to elucidate the role of sex differences and their interactions with gut microbiota in the onset and progression of PD disease.

Some several strengths and limitations of this study need to be acknowledged. Its main advantage is the first-ever exploration of the changes in gut microbiota in PD patients that takes into account patients’ gender and combine it with metagenomics sequencing technology. In addition, for the first time, the fMRI technique was utilized to advance the understanding of the relationship between microbiota changes and brain function. However, our study has some weaknesses: (i) It was designed as a pilot study with a small sample size. (ii) Healthy controls were not included, although previous studies have shown differences in gut microbiotas between PD patients and normal subjects. (iii) The evaluation of the relationship between altered gut microbiota and brain function was limited to association analysis, and more basic research is needed to identify the underlying mechanisms.

## 5. Conclusion

The present study identified the differences in the composition and function of the gut microbiota between male and female PD patients by metagenomic sequencing. fMRI results confirmed that gender differences exist in specific brain regions in PD patients and may be associated with altered gut microbiota. In conclusion, we provide a novel direction to investigate the mechanism of action of the microbiota-gut-brain axis in male and female PD patients.

## Data availability statement

The sequence data presented in this study are deposited in the NCBI Sequence Read Archive (SRA) database, accession number PRJNA985875.

## Ethics statement

The studies involving human participants were reviewed and approved by the Ethics Committee of the Affiliated Huai’an No.1 People’s Hospital with Nanjing Medical University. The patients/participants provided their written informed consent to participate in this study. Written informed consent was obtained from the individual(s) for the publication of any potentially identifiable images or data included in this article.

## Author contributions

MZ wrote the manuscript and analyzed the data. ZZ collected the samples and prepared the manuscript. BY drew and analyzed the pictures. LH and JW analyzed the data. WD and LX reviewed the manuscript. XY, HW, and YF designed the experiment. All authors contributed to the article submission.

## Footnotes

1
http://huttenhower.sph.harvard.edu/lefse/

2
http://rfmri.org/DPARSF

3
http://www.fil.ion.ucl.ac.uk/spm
